# Vitamin D Serum Levels in Patients with Statin-Induced Musculoskeletal Pain

**DOI:** 10.1155/2019/3549402

**Published:** 2019-03-25

**Authors:** Manuela Pennisi, Giuseppe Di Bartolo, Giulia Malaguarnera, Rita Bella, Giuseppe Lanza, Michele Malaguarnera

**Affiliations:** ^1^Spinal Unit, Emergency Hospital Cannizzaro, Catania, Italy; ^2^Research Center “The Great Senescence”, University of Catania, 95100 Catania, Italy; ^3^Department of Medical and Surgical Sciences and Advanced Technologies, University of Catania, Catania, Italy; ^4^Department of Surgery and Medical-Surgical Specialties, University of Catania, Catania, Italy; ^5^Oasi Research Institute-IRCCS, Via Conte Ruggero, 73-94018 Troina, Italy

## Abstract

**Introduction:**

Statin-associated muscle symptoms are common side effects of statin therapy. These symptoms include myopathy, myalgia, and rhabdomyolysis. Vitamin D has been associated with musculoskeletal health; thus, its deficiency may produce detrimental effects in this tissue. Indeed, one symptom of vitamin D deficiency is myalgia, and the normalization of low vitamin D levels can relieve it.

**Patients and Methods:**

This cross-sectional study examined 1210 statin-treated patients to assess vitamin D status. These patients were divided into two groups: 287 with statin-associated muscle symptoms (SAMS) and 923 control patients without SAMS.

**Results:**

We have found a significant association between deficient and insufficient vitamin D status and statin-associated muscle symptoms (SAMS). Vitamin D deficiency (<30 nmol/L) presents 77% (95% C.I. 71.6% to 81.7%) sensitivity and 63.4% (95% C.I. 60.2% to 66.5%) specificity in diagnosing SAMS. Odds ratio analysis showed that this association is moderate-strong both for deficient and for insufficient status.

**Conclusion:**

We found a correlation between vitamin D deficiency and SAMS. Therefore, vitamin D levels may be useful for the diagnosis and management of SAMS.

## 1. Introduction

**Figure 1:**
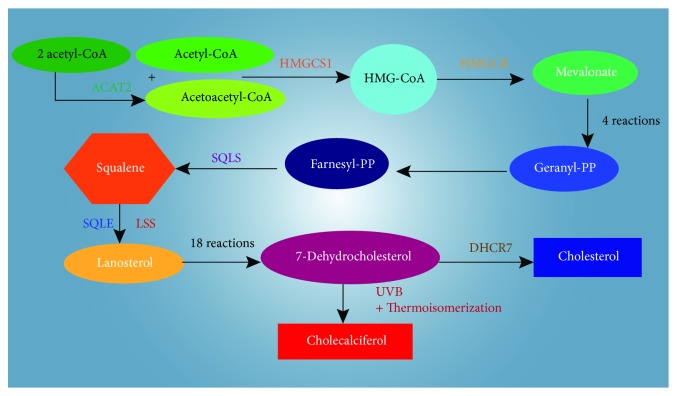
Pathway of cholesterol and cholecalciferol biosynthesis. HMG-CoA: 3-hydroxy-3-methylglutaryl-CoA; PP: pyrophosphate; ACAT2: acetyl-CoA acetyltransferase 2; HMGCS1: HMG-CoA synthase 1; HMGCR: HMG-CoA reductase; FDPS: farnesyl diphosphate synthase; SQLS: squalene synthase; SQLE: squalene; LSS: lanosterol synthase; DHCR7: 7-dehydrocholesterol reductase; UVB: ultraviolet B rays.

Vitamin D is a fat-soluble secosteroid ingested from the diet and produced as vitamin D_3_ in the skin following exposure to ultraviolet rays. It is then converted into its active form in the liver and kidneys [1]. Vitamin D production starts from acetyl-CoA following the cholesterol production pathway until 7-dehydrocholesterol is synthetized (Figure 1). Statins act through the reversible block of the hydroxy-3-methylglutaryl-coenzyme A reductase (HMG-CoAR), thereby reducing cholesterol synthesis and 7-dehydrocholesterol and vitamin D production. Inhibition of HMG-CoAR diminishes also the levels of ubiquinone, steroids, bile acids, geranyl-geranyl pyrophosphate (GGPP), and farnesyl pyrophosphate (FPP) [2]. Although statins are well tolerated, they may produce several side effects, such as muscle weakness, muscle pain or aching (myalgia) stiffness, muscle tenderness, cramps, and arthralgia. These symptoms are defined as statin-associated muscle symptoms (SAMS), and they can manifest with or without an elevation of creatine kinase (CK) serum concentrations. The metabolic processes regulated by vitamin D include serum calcium and phosphate homeostasis, bone remodeling, neuromuscular function, immunity, inflammation, and transcription of proteins involved in cell growth and apoptosis. [3–5]. Vitamin D exerts a clear promyogenic effect on satellite cells responsible for the muscle reconstitution after an injury [6], and it also boosts muscle performance through the increase in size and amount of type II fast twitch fibers, used predominantly in sustained and anaerobic exercise. Exercise-induced tissue damage and lipid peroxidation were significantly lowered by vitamin D treatment in Wistar Kyoto rats [7]. Although serum vitamin D levels influence muscle contractility, strength, and postural stability, there is no consensus about the role of vitamin D status in SAMS. Serum 25OH-vitamin D is the major circulating metabolite of vitamin D in the body and reflects vitamin D inputs from cutaneous synthesis and dietary intake. For this reason, it is considered the standard clinical measure of vitamin D status. The aim of this study was to evaluate the vitamin D status in statin-intolerant patients.

## 2. Patients and Methods

All the participants and control groups were inpatients from January 2010 to December 2016 in the Cannizzaro Hospital, Catania, Italy. SAMS patients are defined by the European Atherosclerosis Society Consensus Panel [8]. We enrolled 1210 hypercholesterolemic patients treated with statins. These patients were divided into two groups: 287 with SAMS and 923 control patients without SAMS. The exclusion criteria were as follows:

1. Subjects treated with vitamin D
2. Subjects treated with corticosteroids
3. Subjects with uncontrolled infectious disease, autoimmune diseases, severe renal dysfunction, history of hepatitis C or positive detection of serum hepatitis B virus antigen, neuropsychiatric disorders, malignancy, and hormone replacement therapy
4. A history of alcohol abuse
5. Subjects in a vegan or vegetarian diet

Conventional risk factors evaluated in this study were history of hypertension, diabetes mellitus, cigarette smoking, and body mass index (reported in Table 1).

We analyzed the serum levels of vitamin D in statin-treated patients with SAMS compared with patients without SAMS. The serum levels were defined as deficient (<30 nmol/L), insufficient (30-50 nmol/L), and sufficient (>50 nmol/L). Subjects with pain and elevation of CK were addressed to the SAMS group.

The study complied with the Declaration of Helsinki and was approved by the Ethics Committee of Cannizzaro Hospital. Written consent was obtained from all participants.

### 2.1. Laboratory Measurements

Venous blood samples were collected after overnight fasting. We used an automatic biochemical analyzer to measure triglyceride serum levels, fasting plasma glucose, creatinine, azotemia, total cholesterol (TC), low-density lipoprotein cholesterol (LDL-C), high-density lipoprotein cholesterol (HDL-C), and total bilirubin.

We measured serum alanine aminotransferase (ALT) and aspartate aminotransferase (AST) using an enzymatic calorimetric test. C-reactive protein (CRP) was measured by the high-sensitivity nephelometric method.

Serum samples were centrifugated at 1500xg for 10 min and stored at 80°C for future measurements of vitamin D levels by an immunoenzymatic assay (Beckman Coulter). Coefficient of variation of intra-assay and interassay was, respectively, 3.2% and 7.1% [9].

### 2.2. Statistics

The results are presented as mean ± standard deviation. The following two-tailed tests were used to evaluate the study: Student's *t*-test was used for comparing means and Wald and chi-square analyses were used to compare categorical variables, which were presented as percentage. We used IBM SPSS for Windows version 23.0 (IBM Corp., Armonk, USA). We analyzed the data obtained and calculated sensitivity, specificity, positive predictive value (PPV), negative predictive value (NPV), positive likelihood ratio (PLR), and negative likelihood ratio (NLR). Odds ratio and 95% confidence interval were calculated to assess associations between risk of vitamin D deficiency and statin intolerance.

## 3. Results

Among 1210 patients, SAMS were present in 287 patients. 122 patients (42.5%) showed a vitamin D deficiency (<30 nmol/L), 99 patients (34.5%) presented an insufficient vitamin D status (30-50 nmol/L), and 66 (23%) displayed a sufficient vitamin D status (>50 nmol/L). Among the 923 patients without SAMS, 104 patients (11.3%) showed 25OH − vitamin D < 30 nmol/L, 235 (25.4%) 25OH-vitamin D between 30 and 50 nmol/L, and 584 (63.2%) 25OH − vitamin D > 50 nmol/L.

In the comparison between SAMS patients and the control group, we observed a significant difference (*P* < 0.05) in BUN, fasting blood glucose, and triglycerides and a highly significant difference in total cholesterol, creatinine, CPK, CRP, and 25OH-vitamin D serum levels (*P* < 0.0001) (Tables 2 and 3). The calculated odds ratio for 30 nmol/L and 50 nmol/L as cutoffs were, respectively, 5.76 and 5.82 showing a moderate/strong association between vitamin D and SAMS under 30 and 50 nmol/L. Moreover, a 30 nmol/L cutoff showed a high sensitivity of 77% and a specificity of 63.47% in finding SAMS. Conversely, a 50 nmol/L cutoff showed an intermediate-low (42.51%) sensitivity and high (88.73%) specificity. We found low positive predictive values for vitamin D < 30 nmol/L and <50 nmol/L (53.98% and 39.46%, respectively) and very high negative predictive value (89.92% and 83.23%, respectively). Multinominal regression analysis considering the vitamin D status as the independent variable and SAMS as the dependent variable showed a strong significant association (*P* < 0.0001) between SAMS and deficient and insufficient statuses of vitamin D (as reported in Table 4).

## 4. Discussion

Statins are reversible competitive inhibitors of 3-hydroxy-3-methylglutaryl-coenzyme A reductase (HMG-CoAR) and consequently reduce intracellular synthesis of cholesterol. Although statins are well tolerated, they may produce several side effects. The evidence suggests that around 40-75% of these patients discontinue their statin therapy within one year after initiation [10]. Unfortunately, this behavior correlates highly with risk for acute cardiovascular events such as recurrent myocardial infarction and coronary heart defect [11]. About half of patients discontinue statin therapy within the first year, and adherence decreases with time probably because of multifactorial and statin-induced muscle symptoms, which are a major reason for the drug discontinuation [12]. Supplementation with carnitine [13, 14], resveratrol [15], silybin [16], silibinin [17], and coenzyme Q10 [18, 19] has shown conflicting results in decreasing SAMS.

We evaluated vitamin D serum levels in 1210 statin-treated patients. Vitamin D serum levels in patients with SAMS were lower (36.1 nmol/L) (*P* < 0.0001) (95% C.I. 32.5 to 39.6). The absence of diagnostic tests requires diagnosis of SAMS on the basis of clinical criteria. A vitamin D deficiency value of <30 nmol/L presents 77% (95% C.I. 71.6% to 81.7%) sensitivity and 63.4% (95% C.I. 60.2% to 66.5%) specificity for SAMS. Moreover, we found a significant association (*P* < 0.0001) between deficient and insufficient vitamin D statuses and the muscular symptoms due to statin therapy. Vitamin D deficiency has been independently associated with muscle weakness and severe myopathy and may, in fact, be a confounder for statin-induced myopathies [12]. A study on ovariectomized rats implied serum vitamin D deficiency in the etiology of deep muscle pain [20]. Another study on rats receiving supplemental vitamin D showed a decrease in plasma creatine kinase levels (CK) and inflammatory cytokines such as IL-6 and TNF [21].

Several studies showed that vitamin D deficiency can lead to an increased susceptibility to the development of SAMS [12, 22–27]. Bischoff-Ferrari et al. had found that every 1 ng/mL decrease in vitamin D levels was associated with an increase of 1.22 times the hazard of SAMS [28]. Moreover, recent research suggests that vitamin D deficiency may impair the lipid response of statins and increase the risk of myopathy in statin users [28]. Kang et al. demonstrated that statin rechallenge in patients who were treated with vitamin D was better tolerated [29]. Nevertheless, other studies deny the relationship between the concentrations of vitamin D and the risk of muscle-related side effects in statin-treated adults [30–34]. The discrepancy between the reported studies is unclear. The physical chemical properties of statin can influence the type and frequency of adverse effects. Short-term studies have shown that more lipophilic statins can cause increases in various metabolites of vitamin D, while less lipophilic statins provide no improvement in vitamin D [10, 12]. It may be due to differences in the population studied, the features of the used statin, the intensity of cholesterol lowering, and the ethnic background of the subjects or to possible related differences in the prevalence of subclinical genetic myopathies, certain single-nucleotide polymorphisms, vitamin D-binding protein genetic variants [35], and CYP3A4 activity [36].

## 5. Conclusion

Our study aligns itself with the data supporting the hypothesis that vitamin D and SAMS are interconnected. Vitamin D status may represent an important tool useful for diagnosis and management of SAMS. Further studies are needed to evaluate the relationship between vitamin D and SAMS.

## Figures and Tables

**Table 1:** Clinical characteristics of the patients.

	SAMS (*N* = 287)	Controls (*N* = 923)	*P*
Age	53	52	/
Age range	45-68	46-67	/
Sex (M/F)	137/150	425/562	/
SBP (mmHg)	141 ± 16.4	142.4 ± 16.5	NS
DBP (mmHg)	80.7 ± 9.4	81.8 ± 7.9	*P* < 0.05
Heart rate (bpM)	84.2 ± 9.7	83.8 ± 9.8	NS
BMI (kg/m^2^)	24.5 ± 2.9	24.6 ± 2.8	NS
Current smokers	97 (33.8%)	482 (52.2%)	*P* < 0.0001
Past smokers	83 (28.9%)	210 (22.7%)	*P* < 0.05
Hypertension	72 (25%)	324 (37%)	*P* < 0.05
Diabetes mellitus	39 (13.6%)	96 (10.4%)	NS

**Table 2:** Comparison of laboratory parameters between SAMS and control patients.

	SAMS (*N* = 287)	Control (*N* = 923)	*P*
Blood urea nitrogen	45.1 ± 5.6	44.2 ± 5.4	*P* < 0.05
Blood glucose (mg/dL)	98.2 ± 31.4	93.4 ± 36.1	*P* < 0.05
Creatinine (mg/dL)	0.94 ± 0.18	0.87 ± 0.24	*P* < 0.0001
Total cholesterol (mg/dL)	265 ± 24.2	271.4 ± 20.1	*P* < 0.0001
LDL-cholesterol (mg/dL)	125.4 ± 21.4	126.9 ± 20.7	NS
HDL-cholesterol (mg/dL)	38.2 ± 12.4	37.4 ± 12.8	NS
Triglycerides (mg/dL)	178.1 ± 27.4	184.2 ± 27.8	*P* < 0.05
CPK (U/L)	54.1 ± 12.4	50.2 ± 12.0	*P* < 0.0001
LDH (U/L)	325.6 ± 44.2	328.1 ± 47.6	NS
CRP (mg/dL)	4.25 ± 0.67	1.87 ± 0.56	*P* < 0.0001
AST (U/L)	34.2 ± 18.2	33.6 ± 17.9	NS
ALT (U/L)	35.4 ± 16.8	35.9 ± 16.1	NS
Total bilirubin (mg/dL)	1.29 ± 0.67	1.32 ± 0.51	NS
Vitamin D (nmol/L)	48.1 ± 21.6	84.2 ± 27.8	*P* < 0.0001

SAMS: statin-associated muscle symptoms; LDL: low-density lipoprotein; HDL: high-density lipoprotein; CPK: creatine phosphokinase; LDH: lactic dehydrogenase; CRP: C-reactive protein; AST: aspartate aminotransferase; ALT: alanine aminotransferase.

**Table 3:** Vitamin D status in statin-treated patients.

Vitamin D status (nmol/L)	287 patients with SAMS		923 control patients		Chi-square
>50 nmol/L	66	23%	584	63.2%	/
30-50 nmol/L	99	34.5%	235	25.4%	(*P* < 0.0001)
<30 nmol/L	122	42.5%	104	11.3%	(*P* < 0.0001)

**Table 4:** Predictive values of vitamin D deficiency and insufficiency.

	<30 nmol/L	95% CI	<50 nmol/L	95% CI
Sensitivity	77.00%	71.69% to 81.74%	42.51%	36.72% to 48.45%
Specificity	63.47%	60.28% to 66.57%	88.73%	86.51% to 90.70%
Positive likelihood ratio	2.11	1.90 to 2.34	3.77	3.01 to 4.73
Negative likelihood ratio	0.36	0.29 to 0.45	0.65	0.59 to 0.72
Disease prevalence	23.62%	21.26% to 26.11%	23.72%	21.35% to 26.22%
Positive predictive value	39.46%	36.97% to 42.02%	53.98%	48.35% to 59.51%
Negative predictive value	89.92%	87.78% to 91.73%	83.23%	81.76% to 84.61%
Accuracy	66.67%	63.94% to 69.32%	77.77%	75.32% to 80.08%
Odds ratio	5.7685	4.2488 to 7.8317	5.8227	4.2686 to 7.9427

## Data Availability

The data used to support the findings of this study are included within the article.

## References

[1] Holick M. F. (2007). Vitamin D deficiency. *The New England Journal of Medicine*.

[2] Brown A. J., Ikonen E., Olkkonen V. M. (2014). Cholesterol precursors: more than mere markers of biosynthesis. *Current Opinion in Lipidology*.

[3] Holick M. F. (2006). High prevalence of vitamin D inadequacy and implications for health. *Mayo Clinic Proceedings*.

[4] Di Rosa M., Malaguarnera G., De Gregorio C., Palumbo M., Nunnari G., Malaguarnera L. (2012). Immuno-modulatory effects of vitamin D3 in human monocyte and macrophages. *Cellular Immunology*.

[5] Di Rosa M., Malaguarnera M., Nicoletti F., Malaguarnera L. (2011). Vitamin D3: a helpful immuno-modulator. *Immunology*.

[6] Braga M., Simmons Z., Norris K. C., Ferrini M. G., Artaza J. N. (2017). Vitamin D induces myogenic differentiation in skeletal muscle derived stem cells. *Endocrine Connections*.

[7] Ke C. Y., Yang F. L., Wu W. T. (2016). Vitamin D_3_ reduces tissue damage and oxidative stress caused by exhaustive exercise. *International Journal of Medical Sciences*.

[8] Stroes E. S., Thompson P. D., Corsini A. (2015). Statin-associated muscle symptoms: impact on statin therapy—European atherosclerosis society consensus panel statement on assessment, aetiology and management. *European Heart Journal*.

[9] Thienpont L. M., Stepman H. C., Vesper H. W. (2012). Standardization of measurements of 25-hydroxyvitamin D3 and D2. *Scandinavian Journal of Clinical and Laboratory Investigation*.

[10] Banach M., Stulc T., Dent R., Toth P. P. (2016). Statin non-adherence and residual cardiovascular risk: there is need for substantial improvement. *International Journal of Cardiology*.

[11] Toth P. P., Patti A. M., Giglio R. V. (2018). Management of statin intolerance in 2018: still more questions than answers. *American Journal of Cardiovascular Drugs*.

[12] Riche K. D., Arnall J., Rieser K., East H. E., Riche D. M. (2016). Impact of vitamin D status on statin-induced myopathy. *Journal of Clinical & Translational Endocrinology*.

[13] Malaguarnera M., Vacante M., Motta M., Malaguarnera M., Li Volti G., Galvano F. (2009). Effect of l-carnitine on the size of low-density lipoprotein particles in type 2 diabetes mellitus patients treated with simvastatin. *Metabolism*.

[14] Galvano F., Li Volti G., Malaguarnera M. (2009). Effects of simvastatin and carnitine versus simvastatin on lipoprotein(a) and apoprotein(a) in type 2 diabetes mellitus. *Expert Opinion on Pharmacotherapy*.

[15] Malaguarnera G., Pennisi M., Bertino G. (2018). Resveratrol in patients with minimal hepatic encephalopathy. *Nutrients*.

[16] Malaguarnera M., Motta M., Vacante M. (2015). Silybin-vitamin E-phospholipids complex reduces liver fibrosis in patients with chronic hepatitis C treated with pegylated interferon *α* and ribavirin. *American Journal of Translational Research*.

[17] Marrazzo G., Bosco P., la Delia F. (2011). Neuroprotective effect of silibinin in diabetic mice. *Neuroscience Letters*.

[18] Taylor B. A., Lorson L., White C. M., Thompson P. D. (2015). A randomized trial of coenzyme Q10 in patients with confirmed statin myopathy. *Atherosclerosis*.

[19] Skarlovnik A., Janić M., Lunder M., Turk M., Šabovič M. (2014). Coenzyme Q10 supplementation decreases statin-related mild-to-moderate muscle symptoms: a randomized clinical study. *Medical Science Monitor*.

[20] Tague S. E., Clarke G. L., Winter M. K., McCarson K. E., Wright D. E., Smith P. G. (2011). Vitamin D deficiency promotes skeletal muscle hypersensitivity and sensory hyperinnervation. *The Journal of Neuroscience*.

[21] Choi M., Park H., Cho S., Lee M. (2013). Vitamin D_3_ supplementation modulates inflammatory responses from the muscle damage induced by high-intensity exercise in SD rats. *Cytokine*.

[22] Ahmed W., Khan N., Glueck C. J. (2009). Low serum 25 (OH) vitamin D levels (<32 ng/mL) are associated with reversible myositis-myalgia in statin-treated patients. *Translational Research*.

[23] Glueck C. J., Lee K., Prince M., Milgrom A., Makadia F., Wang P. (2017). Low serum vitamin D, statin associated muscle symptoms, vitamin D supplementation. *Atherosclerosis*.

[24] Mergenhagen K., Ott M., Heckman K., Rubin L. M., Kellick K. (2014). Low vitamin D as a risk factor for the development of myalgia in patients taking high-dose simvastatin: a retrospective review. *Clinical Therapeutics*.

[25] Shantha G. P. S., Ramos J., Thomas-Hemak L., Pancholy S. B. (2014). Association of vitamin D and incident statin induced myalgia—a retrospective cohort study. *PLoS One*.

[26] Minissian M., Agarwal M., Shufelt C. (2015). Do women with statin-related myalgias have low vitamin D levels?. *BMC Research Notes*.

[27] Michalska-Kasiczak M., Sahebkar A., Mikhailidis D. P. (2015). Analysis of vitamin D levels in patients with and without statin-associated myalgia — a systematic review and meta-analysis of 7 studies with 2420 patients. *International Journal of Cardiology*.

[28] Bischoff-Ferrari H. A., Fischer K., Orav E. J. (2017). Statin use and 25-hydroxyvitamin D blood level response to vitamin D treatment of older adults. *Journal of the American Geriatrics Society*.

[29] Kang J. H., Nguyen Q. N., Mutka J., le Q. A. (2017). Rechallenging statin therapy in veterans with statin-induced myopathy post vitamin D replenishment. *Journal of Pharmacy Practice*.

[30] Khayznikov M., Hemachrandra K., Pandit R., Kumar A., Wang P., Glueck C. J. (2015). Statin intolerance because of myalgia, myositis, myopathy, or myonecrosis can in most cases be safely resolved by vitamin d supplementation. *North American Journal of Medical Sciences*.

[31] Eisen A., Lev E., Iakobishvilli Z. (2014). Low plasma vitamin D levels and muscle-related adverse effects in statin users. *The Israel Medical Association Journal*.

[32] Kurnik D., Hochman I., Vesterman-Landes J. (2012). Muscle pain and serum creatine kinase are not associated with low serum 25(OH) vitamin D levels in patients receiving statins. *Clinical Endocrinology*.

[33] Riphagen I. J., van der Veer E., Muskiet F. A. J., DeJongste M. J. L. (2012). Myopathy during statin therapy in the daily practice of an outpatient cardiology clinic: prevalence, predictors and relation with vitamin D. *Current Medical Research and Opinion*.

[34] Backes J. M., Barnes B. J., Ruisinger J. F., Moriarty P. M. (2011). A comparison of 25-hydroxyvitamin D serum levels among those with or without statin-associated myalgias. *Atherosclerosis*.

[35] Lauridsen A. L., Vestergaard P., Hermann A. P. (2005). Plasma concentrations of 25-hydroxy-vitamin D and 1,25-dihydroxy-vitamin D are related to the phenotype of Gc (vitamin D-binding protein): a cross-sectional study on 595 early postmenopausal women. *Calcified Tissue International*.

[36] Gupta R. P., He Y. A., Patrick K. S., Halpert J. R., Bell N. H. (2005). CYP3A4 is a vitamin D-24- and 25-hydroxylase: analysis of structure function by site-directed mutagenesis. *The Journal of Clinical Endocrinology and Metabolism*.

